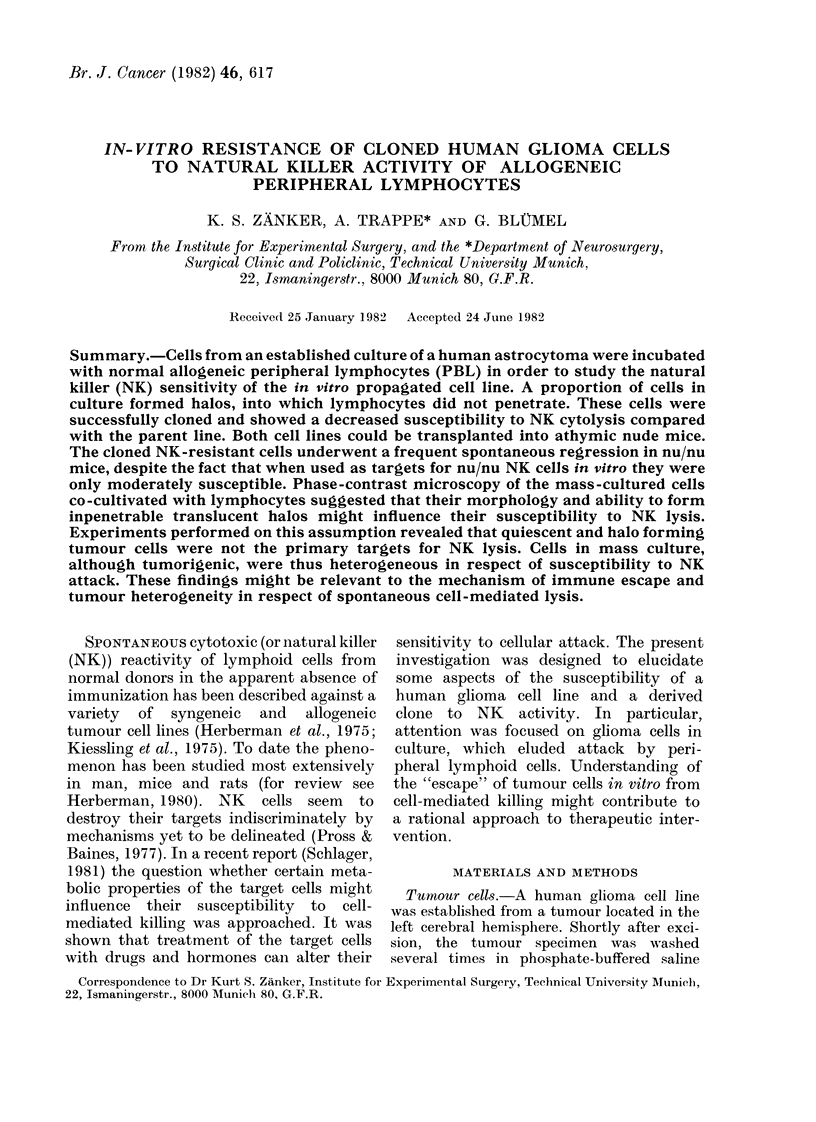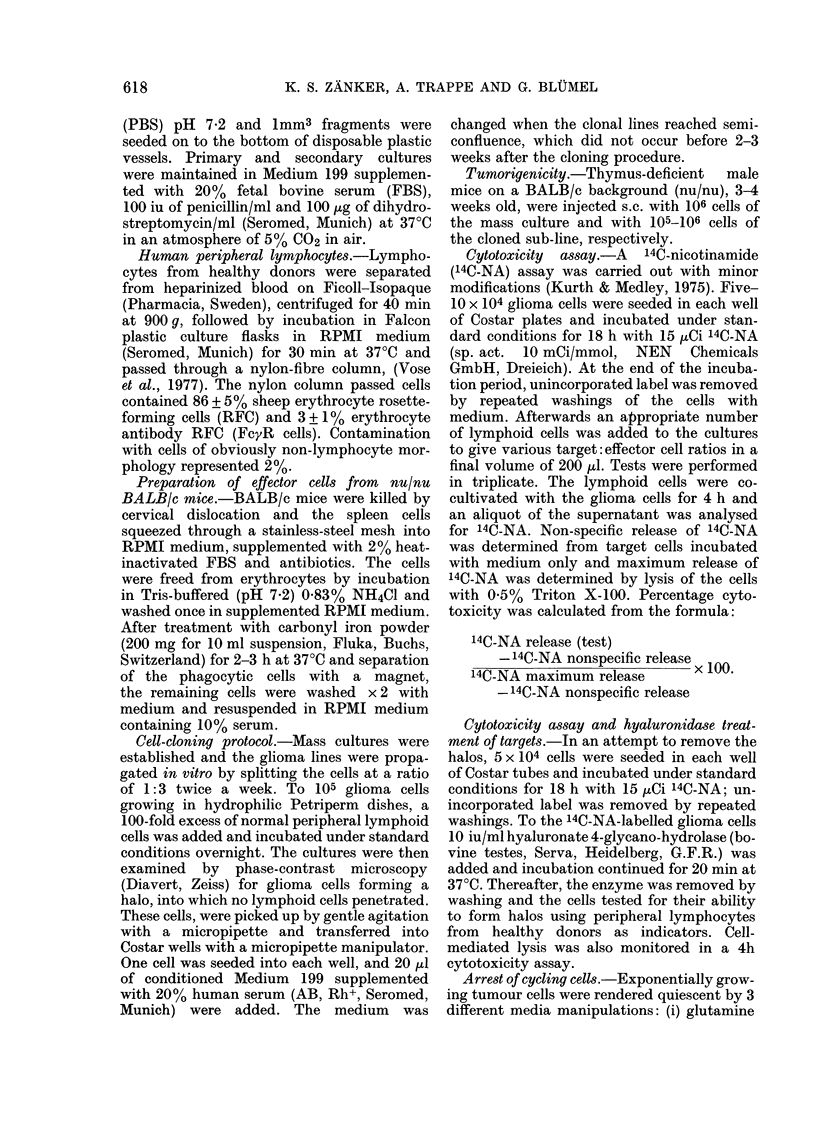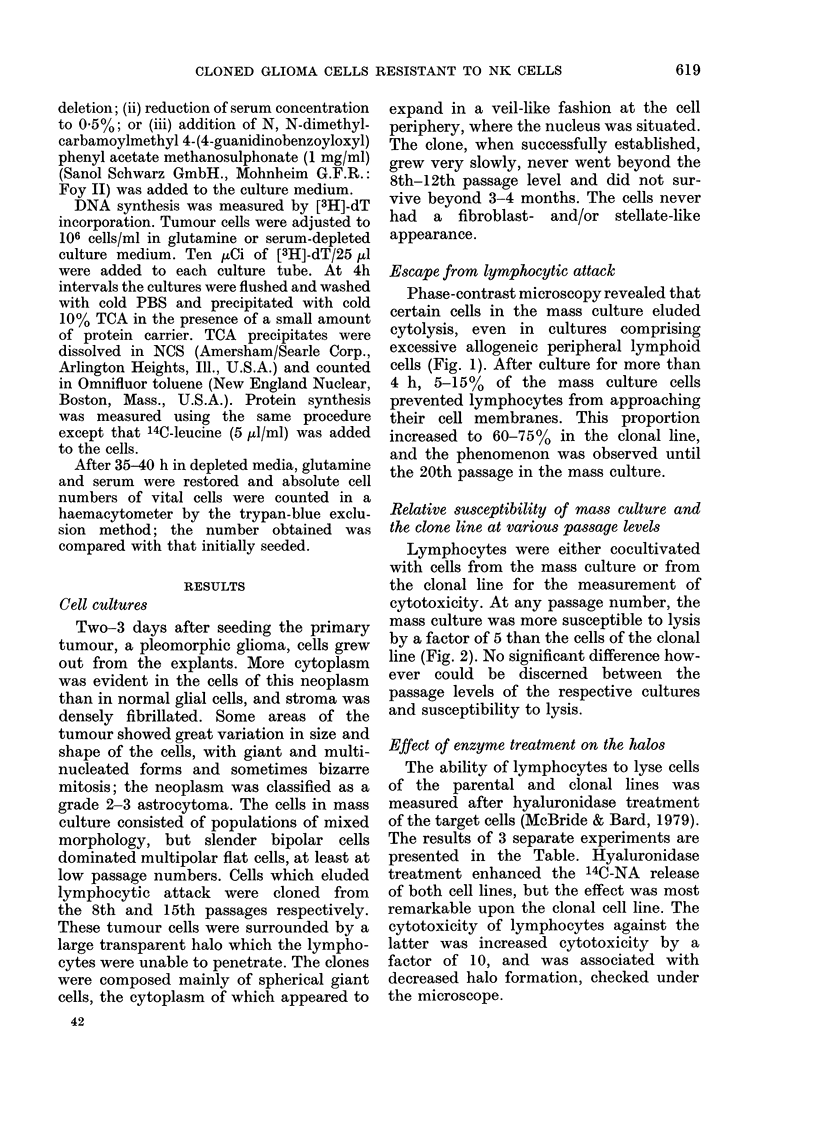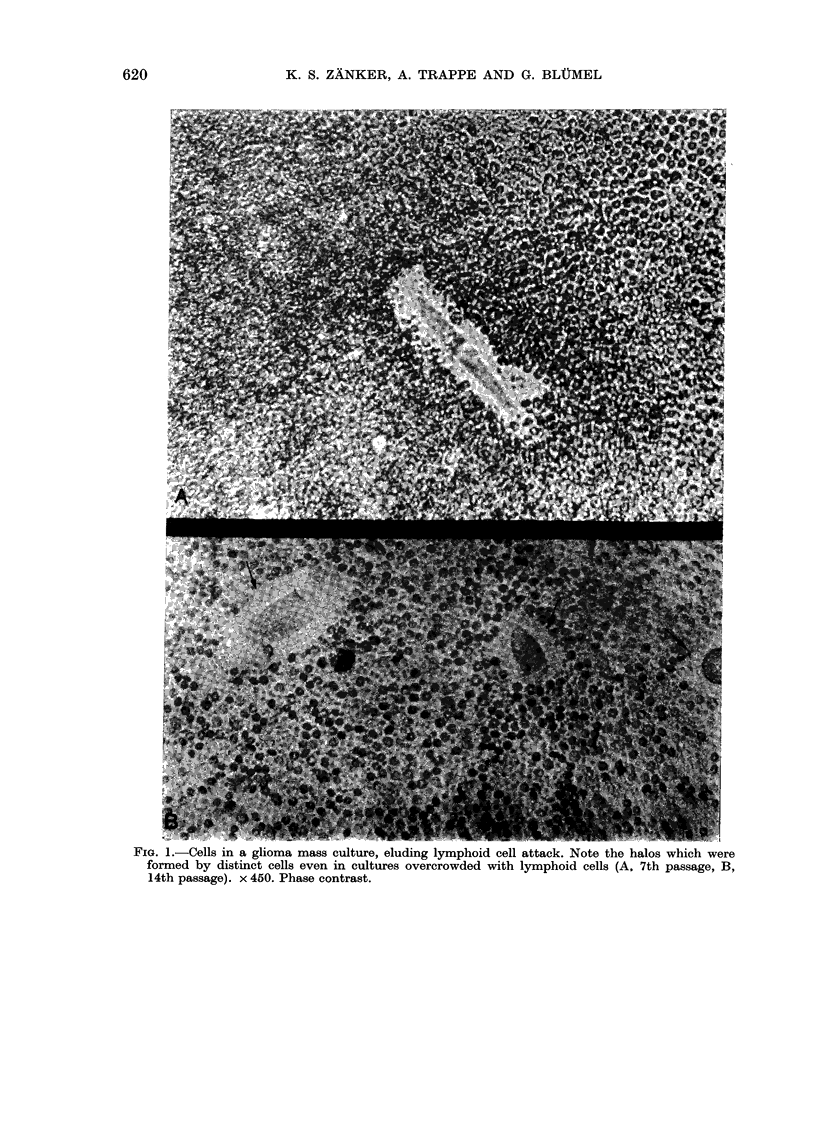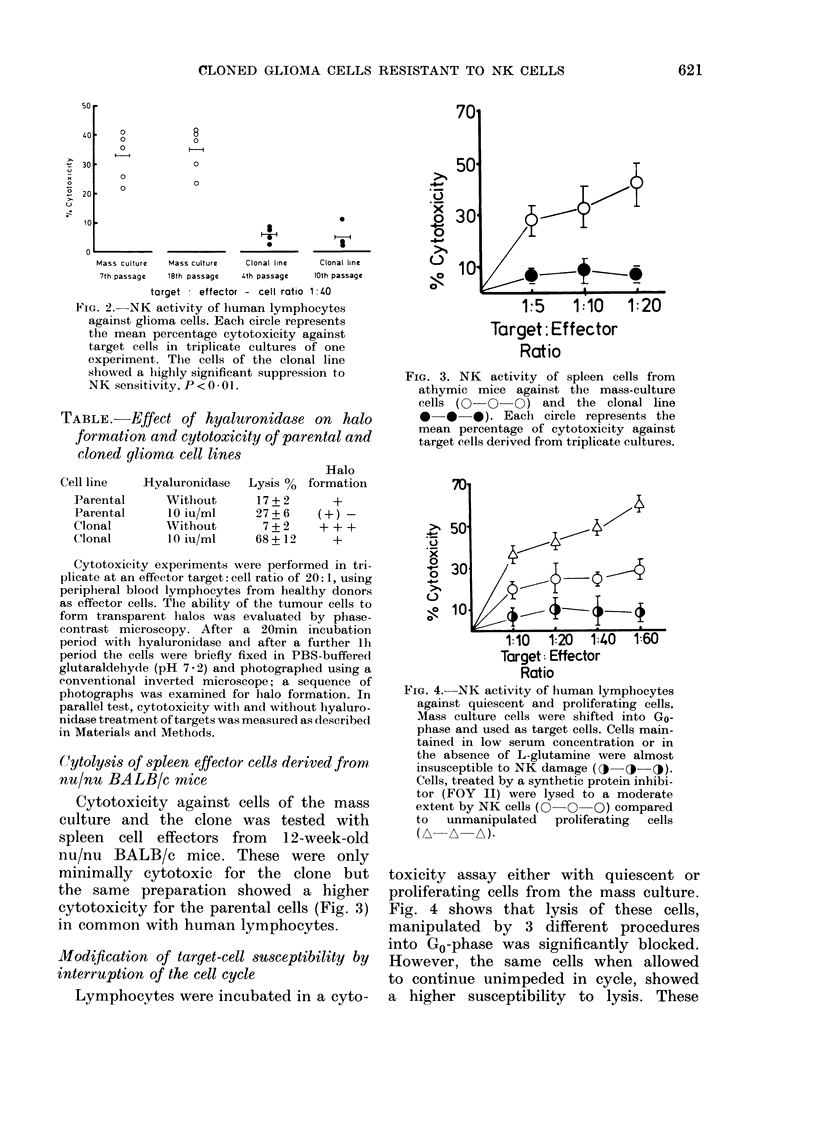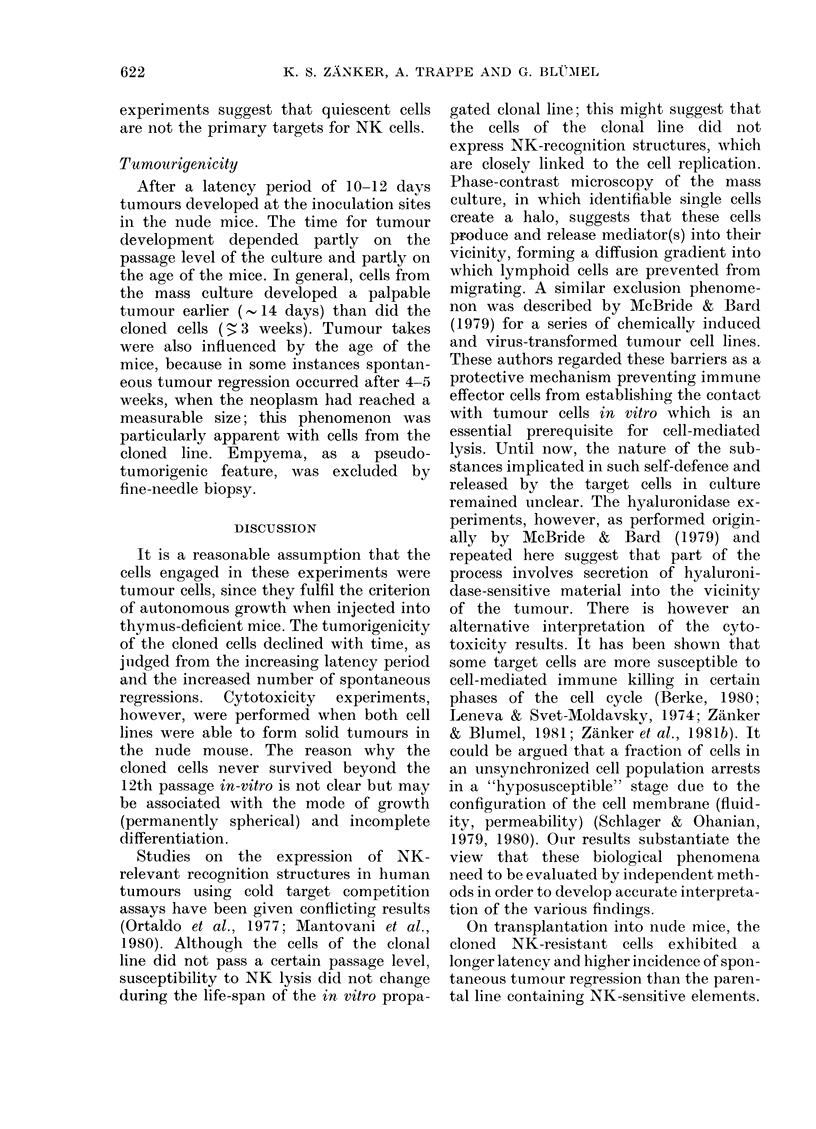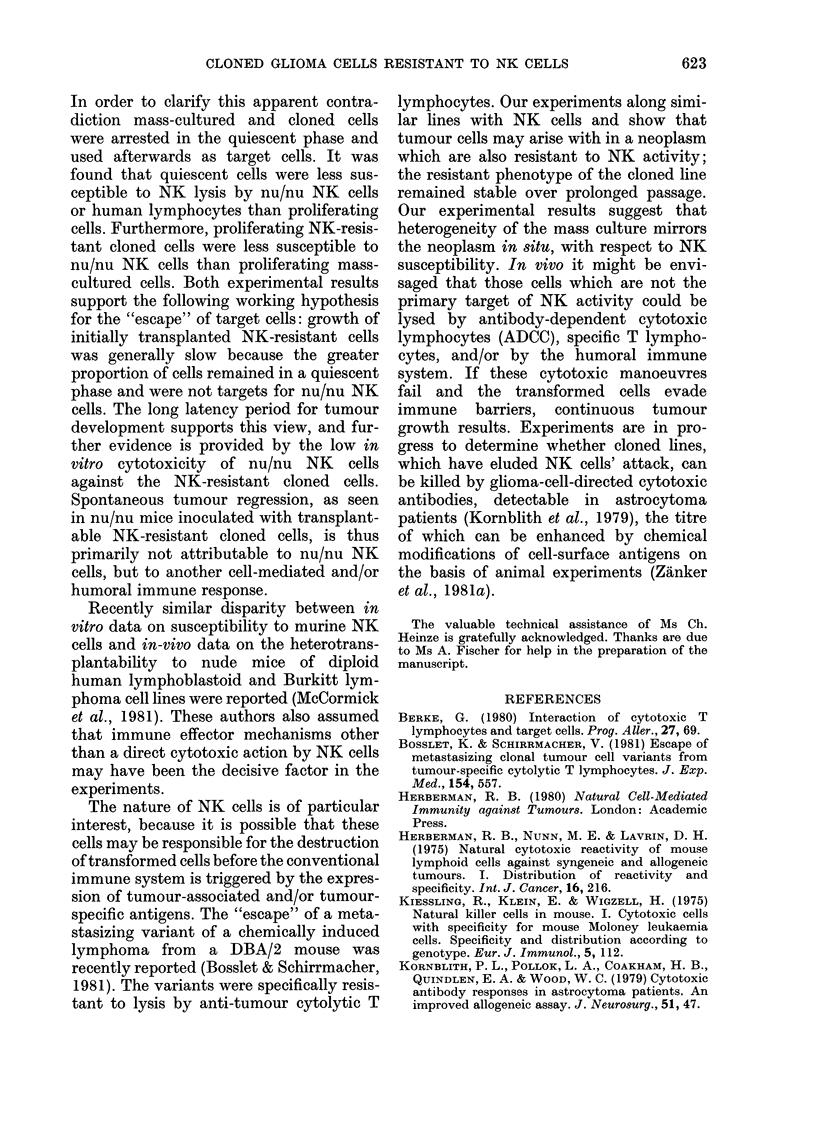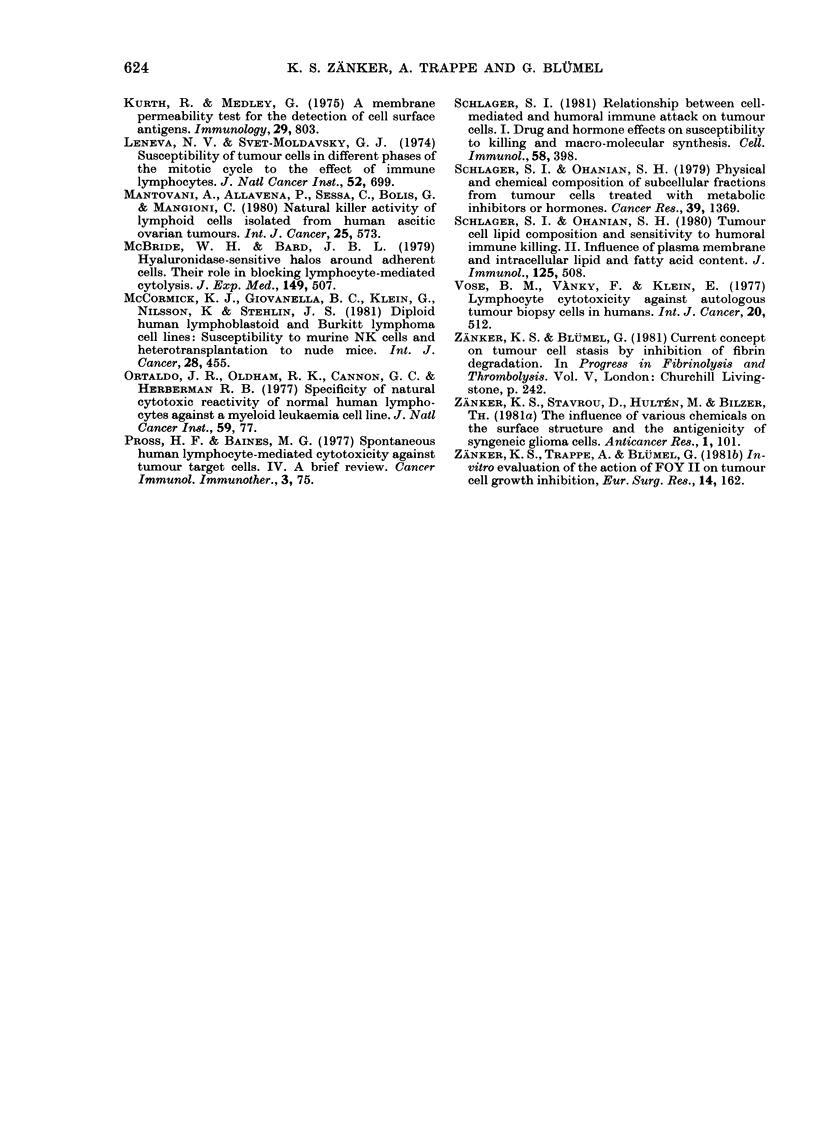# In-vitro resistance of cloned human glioma cells to natural killer activity of allogeneic peripheral lymphocytes.

**DOI:** 10.1038/bjc.1982.246

**Published:** 1982-10

**Authors:** K. S. Zänker, A. Trappe, G. Blümel

## Abstract

**Images:**


Br. J. Cancer (1982) 46, 617

### *IN-VITRO* RESISTANCE OF CLONED HUMAN GLIOMA CELLS TO NATURAL KILLER ACTIVITY OF ALLOGENEIC PERIPHERAL LYMPHOCYTES

K. S. ZÄNKER, A. TRAPPE* AND G. BLÜMEL

*From the Institute for Experimental Surgery, and the *Department of Neurosurgery, Surgical Clinic and Policlinic, Technical University Munich, 22, Ismaningerstr., 8000 Munich 80, G.F.R.*

Received 25 January 1982 Accepted 24 June 1982

**Summary.—Cells from an established culture of a human astrocytoma were incubated with normal allogeneic peripheral lymphocytes (PBL) in order to study the natural killer (NK) sensitivity of the *in vitro* propagated cell line. A proportion of cells in culture formed halos, into which lymphocytes did not penetrate. These cells were successfully cloned and showed a decreased susceptibility to NK cytolysis compared with the parent line. Both cell lines could be transplanted into athymic nude mice. The cloned NK-resistant cells underwent a frequent spontaneous regression in nu/nu mice, despite the fact that when used as targets for nu/nu NK cells *in vitro* they were only moderately susceptible. Phase-contrast microscopy of the mass-cultured cells co-cultivated with lymphocytes suggested that their morphology and ability to form inpenetrable translucent halos might influence their susceptibility to NK lysis. Experiments performed on this assumption revealed that quiescent and halo forming tumour cells were not the primary targets for NK lysis. Cells in mass culture, although tumorigenic, were thus heterogeneous in respect of susceptibility to NK attack. These findings might be relevant to the mechanism of immune escape and tumour heterogeneity in respect of spontaneous cell-mediated lysis.**

SPONTANEOUS cytotoxic (or natural killer (NK)) reactivity of lymphoid cells from normal donors in the apparent absence of immunization has been described against a variety of syngeneic and allogeneic tumour cell lines (Herberman *et al.*, 1975; Kiessling *et al.*, 1975). To date the phenomenon has been studied most extensively in man, mice and rats (for review see Herberman, 1980). NK cells seem to destroy their targets indiscriminately by mechanisms yet to be delineated (Pross & Baines, 1977). In a recent report (Schlager, 1981) the question whether certain metabolic properties of the target cells might influence their susceptibility to cell-mediated killing was approached. It was shown that treatment of the target cells with drugs and hormones can alter their sensitivity to cellular attack. The present investigation was designed to elucidate some aspects of the susceptibility of a human glioma cell line and a derived clone to NK activity. In particular, attention was focused on glioma cells in culture, which eluded attack by peripheral lymphoid cells. Understanding of the "escape" of tumour cells *in vitro* from cell-mediated killing might contribute to a rational approach to therapeutic intervention.

### MATERIALS AND METHODS

*Tumour cells.*—A human glioma cell line was established from a tumour located in the left cerebral hemisphere. Shortly after excision, the tumour specimen was washed several times in phosphate-buffered saline

Correspondence to Dr Kurt S. Zänker, Institute for Experimental Surgery, Technical University Munich, 22, Ismaningerstr., 8000 Munich 80, G.F.R.

(PBS) pH 7·2 and 1mm$^3$ fragments were seeded on to the bottom of disposable plastic vessels. Primary and secondary cultures were maintained in Medium 199 supplemented with 20% fetal bovine serum (FBS), 100 iu of penicillin/ml and 100 $\mu$g of dihydrostreptomycin/ml (Seromed, Munich) at 37°C in an atmosphere of 5% $CO_2$ in air.

*Human peripheral lymphocytes.*—Lymphocytes from healthy donors were separated from heparinized blood on Ficoll–Isopaque (Pharmacia, Sweden), centrifuged for 40 min at 900 *g*, followed by incubation in Falcon plastic culture flasks in RPMI medium (Seromed, Munich) for 30 min at 37°C and passed through a nylon-fibre column, (Vose *et al.*, 1977). The nylon column passed cells contained $86 \pm 5\%$ sheep erythrocyte rosette-forming cells (RFC) and $3 \pm 1\%$ erythrocyte antibody RFC (Fc$\gamma$R cells). Contamination with cells of obviously non-lymphocyte morphology represented 2%.

*Preparation of effector cells from nu/nu BALB/c mice.*—BALB/c mice were killed by cervical dislocation and the spleen cells squeezed through a stainless-steel mesh into RPMI medium, supplemented with 2% heat-inactivated FBS and antibiotics. The cells were freed from erythrocytes by incubation in Tris-buffered (pH 7·2) 0·83% $NH_4Cl$ and washed once in supplemented RPMI medium. After treatment with carbonyl iron powder (200 mg for 10 ml suspension, Fluka, Buchs, Switzerland) for 2–3 h at 37°C and separation of the phagocytic cells with a magnet, the remaining cells were washed ×2 with medium and resuspended in RPMI medium containing 10% serum.

*Cell-cloning protocol.*—Mass cultures were established and the glioma lines were propagated *in vitro* by splitting the cells at a ratio of 1:3 twice a week. To $10^5$ glioma cells growing in hydrophilic Petriperm dishes, a 100-fold excess of normal peripheral lymphoid cells was added and incubated under standard conditions overnight. The cultures were then examined by phase-contrast microscopy (Diavert, Zeiss) for glioma cells forming a halo, into which no lymphoid cells penetrated. These cells, were picked up by gentle agitation with a micropipette and transferred into Costar wells with a micropipette manipulator. One cell was seeded into each well, and 20 $\mu$l of conditioned Medium 199 supplemented with 20% human serum (AB, Rh$^+$, Seromed, Munich) were added. The medium was changed when the clonal lines reached semi-confluence, which did not occur before 2–3 weeks after the cloning procedure.

*Tumorigenicity.*—Thymus-deficient male mice on a BALB/c background (nu/nu), 3–4 weeks old, were injected s.c. with $10^6$ cells of the mass culture and with $10^5$–$10^6$ cells of the cloned sub-line, respectively.

*Cytotoxicity assay.*—A $^{14}$C-nicotinamide ($^{14}$C-NA) assay was carried out with minor modifications (Kurth & Medley, 1975). Five–$10 \times 10^4$ glioma cells were seeded in each well of Costar plates and incubated under standard conditions for 18 h with 15 $\mu$Ci $^{14}$C-NA (sp. act. 10 mCi/mmol, NEN Chemicals GmbH, Dreieich). At the end of the incubation period, unincorporated label was removed by repeated washings of the cells with medium. Afterwards an appropriate number of lymphoid cells was added to the cultures to give various target:effector cell ratios in a final volume of 200 $\mu$l. Tests were performed in triplicate. The lymphoid cells were co-cultivated with the glioma cells for 4 h and an aliquot of the supernatant was analysed for $^{14}$C-NA. Non-specific release of $^{14}$C-NA was determined from target cells incubated with medium only and maximum release of $^{14}$C-NA was determined by lysis of the cells with 0·5% Triton X-100. Percentage cytotoxicity was calculated from the formula:

$$\frac{^{14}\text{C-NA release (test)} - {}^{14}\text{C-NA nonspecific release}}{^{14}\text{C-NA maximum release} - {}^{14}\text{C-NA nonspecific release}} \times 100.$$

*Cytotoxicity assay and hyaluronidase treatment of targets.*—In an attempt to remove the halos, $5 \times 10^4$ cells were seeded in each well of Costar tubes and incubated under standard conditions for 18 h with 15 $\mu$Ci $^{14}$C-NA; unincorporated label was removed by repeated washings. To the $^{14}$C-NA-labelled glioma cells 10 iu/ml hyaluronate 4-glycano-hydrolase (bovine testes, Serva, Heidelberg, G.F.R.) was added and incubation continued for 20 min at 37°C. Thereafter, the enzyme was removed by washing and the cells tested for their ability to form halos using peripheral lymphocytes from healthy donors as indicators. Cell-mediated lysis was also monitored in a 4h cytotoxicity assay.

*Arrest of cycling cells.*—Exponentially growing tumour cells were rendered quiescent by 3 different media manipulations: (i) glutamine deletion; (ii) reduction of serum concentration to 0·5%; or (iii) addition of N, N-dimethylcarbamoylmethyl 4-(4-guanidinobenzoyloxyl) phenyl acetate methanosulphonate (1 mg/ml) (Sanol Schwarz GmbH., Mohnheim G.F.R.: Foy II) was added to the culture medium.

DNA synthesis was measured by [$^3$H]-dT incorporation. Tumour cells were adjusted to $10^6$ cells/ml in glutamine or serum-depleted culture medium. Ten $\mu$Ci of [$^3$H]-dT/25 $\mu$l were added to each culture tube. At 4h intervals the cultures were flushed and washed with cold PBS and precipitated with cold 10% TCA in the presence of a small amount of protein carrier. TCA precipitates were dissolved in NCS (Amersham/Searle Corp., Arlington Heights, Ill., U.S.A.) and counted in Omnifluor toluene (New England Nuclear, Boston, Mass., U.S.A.). Protein synthesis was measured using the same procedure except that $^{14}$C-leucine (5 $\mu$l/ml) was added to the cells.

After 35–40 h in depleted media, glutamine and serum were restored and absolute cell numbers of vital cells were counted in a haemacytometer by the trypan-blue exclusion method; the number obtained was compared with that initially seeded.

### RESULTS

### *Cell cultures*

Two–3 days after seeding the primary tumour, a pleomorphic glioma, cells grew out from the explants. More cytoplasm was evident in the cells of this neoplasm than in normal glial cells, and stroma was densely fibrillated. Some areas of the tumour showed great variation in size and shape of the cells, with giant and multinucleated forms and sometimes bizarre mitosis; the neoplasm was classified as a grade 2–3 astrocytoma. The cells in mass culture consisted of populations of mixed morphology, but slender bipolar cells dominated multipolar flat cells, at least at low passage numbers. Cells which eluded lymphocytic attack were cloned from the 8th and 15th passages respectively. These tumour cells were surrounded by a large transparent halo which the lymphocytes were unable to penetrate. The clones were composed mainly of spherical giant cells, the cytoplasm of which appeared to expand in a veil-like fashion at the cell periphery, where the nucleus was situated. The clone, when successfully established, grew very slowly, never went beyond the 8th–12th passage level and did not survive beyond 3–4 months. The cells never had a fibroblast- and/or stellate-like appearance.

### *Escape from lymphocytic attack*

Phase-contrast microscopy revealed that certain cells in the mass culture eluded cytolysis, even in cultures comprising excessive allogeneic peripheral lymphoid cells (Fig. 1). After culture for more than 4 h, 5–15% of the mass culture cells prevented lymphocytes from approaching their cell membranes. This proportion increased to 60–75% in the clonal line, and the phenomenon was observed until the 20th passage in the mass culture.

### *Relative susceptibility of mass culture and the clone line at various passage levels*

Lymphocytes were either cocultivated with cells from the mass culture or from the clonal line for the measurement of cytotoxicity. At any passage number, the mass culture was more susceptible to lysis by a factor of 5 than the cells of the clonal line (Fig. 2). No significant difference however could be discerned between the passage levels of the respective cultures and susceptibility to lysis.

### *Effect of enzyme treatment on the halos*

The ability of lymphocytes to lyse cells of the parental and clonal lines was measured after hyaluronidase treatment of the target cells (McBride & Bard, 1979). The results of 3 separate experiments are presented in the Table. Hyaluronidase treatment enhanced the $^{14}$C-NA release of both cell lines, but the effect was most remarkable upon the clonal cell line. The cytotoxicity of lymphocytes against the latter was increased cytotoxicity by a factor of 10, and was associated with decreased halo formation, checked under the microscope.

FIG. 1.—Cells in a glioma mass culture, eluding lymphoid cell attack. Note the halos which were formed by distinct cells even in cultures overcrowded with lymphoid cells (A, 7th passage, B, 14th passage). ×450. Phase contrast.

FIG. 2.—NK activity of human lymphocytes against glioma cells. Each circle represents the mean percentage cytotoxicity against target cells in triplicate cultures of one experiment. The cells of the clonal line showed a highly significant suppression to NK sensitivity, $P < 0 \cdot 01$.

TABLE.—*Effect of hyaluronidase on halo formation and cytotoxicity of parental and cloned glioma cell lines*

| Cell line | Hyaluronidase | Lysis % | Halo formation |
|---|---|---|---|
| Parental | Without | 17 ± 2 | + |
| Parental | 10 iu/ml | 27 ± 6 | (+) − |
| Clonal | Without | 7 ± 2 | + + + |
| Clonal | 10 iu/ml | 68 ± 12 | + |

Cytotoxicity experiments were performed in triplicate at an effector target: cell ratio of 20:1, using peripheral blood lymphocytes from healthy donors as effector cells. The ability of the tumour cells to form transparent halos was evaluated by phase-contrast microscopy. After a 20min incubation period with hyaluronidase and after a further 1h period the cells were briefly fixed in PBS-buffered glutaraldehyde (pH 7·2) and photographed using a conventional inverted microscope; a sequence of photographs was examined for halo formation. In parallel test, cytotoxicity with and without hyaluronidase treatment of targets was measured as described in Materials and Methods.

*Cytolysis of spleen effector cells derived from nu/nu BALB/c mice*

Cytotoxicity against cells of the mass culture and the clone was tested with spleen cell effectors from 12-week-old nu/nu BALB/c mice. These were only minimally cytotoxic for the clone but the same preparation showed a higher cytotoxicity for the parental cells (Fig. 3) in common with human lymphocytes.

*Modification of target-cell susceptibility by interruption of the cell cycle*

Lymphocytes were incubated in a cytotoxicity assay either with quiescent or proliferating cells from the mass culture. Fig. 4 shows that lysis of these cells, manipulated by 3 different procedures into $G_0$-phase was significantly blocked. However, the same cells when allowed to continue unimpeded in cycle, showed a higher susceptibility to lysis. These

FIG. 3. NK activity of spleen cells from athymic mice against the mass-culture cells (○—○—○) and the clonal line ●—●—●). Each circle represents the mean percentage of cytotoxicity against target cells derived from triplicate cultures.

FIG. 4.—NK activity of human lymphocytes against quiescent and proliferating cells. Mass culture cells were shifted into $G_0$-phase and used as target cells. Cells maintained in low serum concentration or in the absence of L-glutamine were almost insusceptible to NK damage (◑—◑—◑). Cells, treated by a synthetic protein inhibitor (FOY II) were lysed to a moderate extent by NK cells (○—○—○) compared to unmanipulated proliferating cells (△—△—△).

experiments suggest that quiescent cells are not the primary targets for NK cells.

*Tumourigenicity*

After a latency period of 10–12 days tumours developed at the inoculation sites in the nude mice. The time for tumour development depended partly on the passage level of the culture and partly on the age of the mice. In general, cells from the mass culture developed a palpable tumour earlier ($\sim$14 days) than did the cloned cells ($\gtrsim$3 weeks). Tumour takes were also influenced by the age of the mice, because in some instances spontaneous tumour regression occurred after 4–5 weeks, when the neoplasm had reached a measurable size; this phenomenon was particularly apparent with cells from the cloned line. Empyema, as a pseudo-tumorigenic feature, was excluded by fine-needle biopsy.

### DISCUSSION

It is a reasonable assumption that the cells engaged in these experiments were tumour cells, since they fulfil the criterion of autonomous growth when injected into thymus-deficient mice. The tumorigenicity of the cloned cells declined with time, as judged from the increasing latency period and the increased number of spontaneous regressions. Cytotoxicity experiments, however, were performed when both cell lines were able to form solid tumours in the nude mouse. The reason why the cloned cells never survived beyond the 12th passage *in-vitro* is not clear but may be associated with the mode of growth (permanently spherical) and incomplete differentiation.

Studies on the expression of NK-relevant recognition structures in human tumours using cold target competition assays have been given conflicting results (Ortaldo *et al.*, 1977; Mantovani *et al.*, 1980). Although the cells of the clonal line did not pass a certain passage level, susceptibility to NK lysis did not change during the life-span of the *in vitro* propagated clonal line; this might suggest that the cells of the clonal line did not express NK-recognition structures, which are closely linked to the cell replication. Phase-contrast microscopy of the mass culture, in which identifiable single cells create a halo, suggests that these cells produce and release mediator(s) into their vicinity, forming a diffusion gradient into which lymphoid cells are prevented from migrating. A similar exclusion phenomenon was described by McBride & Bard (1979) for a series of chemically induced and virus-transformed tumour cell lines. These authors regarded these barriers as a protective mechanism preventing immune effector cells from establishing the contact with tumour cells *in vitro* which is an essential prerequisite for cell-mediated lysis. Until now, the nature of the substances implicated in such self-defence and released by the target cells in culture remained unclear. The hyaluronidase experiments, however, as performed originally by McBride & Bard (1979) and repeated here suggest that part of the process involves secretion of hyaluronidase-sensitive material into the vicinity of the tumour. There is however an alternative interpretation of the cytotoxicity results. It has been shown that some target cells are more susceptible to cell-mediated immune killing in certain phases of the cell cycle (Berke, 1980; Leneva & Svet-Moldavsky, 1974; Zänker & Blumel, 1981; Zänker *et al.*, 1981*b*). It could be argued that a fraction of cells in an unsynchronized cell population arrests in a "hyposusceptible" stage due to the configuration of the cell membrane (fluidity, permeability) (Schlager & Ohanian, 1979, 1980). Our results substantiate the view that these biological phenomena need to be evaluated by independent methods in order to develop accurate interpretation of the various findings.

On transplantation into nude mice, the cloned NK-resistant cells exhibited a longer latency and higher incidence of spontaneous tumour regression than the parental line containing NK-sensitive elements.

In order to clarify this apparent contradiction mass-cultured and cloned cells were arrested in the quiescent phase and used afterwards as target cells. It was found that quiescent cells were less susceptible to NK lysis by nu/nu NK cells or human lymphocytes than proliferating cells. Furthermore, proliferating NK-resistant cloned cells were less susceptible to nu/nu NK cells than proliferating mass-cultured cells. Both experimental results support the following working hypothesis for the "escape" of target cells: growth of initially transplanted NK-resistant cells was generally slow because the greater proportion of cells remained in a quiescent phase and were not targets for nu/nu NK cells. The long latency period for tumour development supports this view, and further evidence is provided by the low *in vitro* cytotoxicity of nu/nu NK cells against the NK-resistant cloned cells. Spontaneous tumour regression, as seen in nu/nu mice inoculated with transplantable NK-resistant cloned cells, is thus primarily not attributable to nu/nu NK cells, but to another cell-mediated and/or humoral immune response.

Recently similar disparity between *in vitro* data on susceptibility to murine NK cells and *in-vivo* data on the heterotransplantability to nude mice of diploid human lymphoblastoid and Burkitt lymphoma cell lines were reported (McCormick *et al.*, 1981). These authors also assumed that immune effector mechanisms other than a direct cytotoxic action by NK cells may have been the decisive factor in the experiments.

The nature of NK cells is of particular interest, because it is possible that these cells may be responsible for the destruction of transformed cells before the conventional immune system is triggered by the expression of tumour-associated and/or tumour-specific antigens. The "escape" of a metastasizing variant of a chemically induced lymphoma from a DBA/2 mouse was recently reported (Bosslet & Schirrmacher, 1981). The variants were specifically resistant to lysis by anti-tumour cytolytic T lymphocytes. Our experiments along similar lines with NK cells and show that tumour cells may arise with in a neoplasm which are also resistant to NK activity; the resistant phenotype of the cloned line remained stable over prolonged passage. Our experimental results suggest that heterogeneity of the mass culture mirrors the neoplasm *in situ*, with respect to NK susceptibility. *In vivo* it might be envisaged that those cells which are not the primary target of NK activity could be lysed by antibody-dependent cytotoxic lymphocytes (ADCC), specific T lymphocytes, and/or by the humoral immune system. If these cytotoxic manoeuvres fail and the transformed cells evade immune barriers, continuous tumour growth results. Experiments are in progress to determine whether cloned lines, which have eluded NK cells' attack, can be killed by glioma-cell-directed cytotoxic antibodies, detectable in astrocytoma patients (Kornblith *et al.*, 1979), the titre of which can be enhanced by chemical modifications of cell-surface antigens on the basis of animal experiments (Zänker *et al.*, 1981*a*).

The valuable technical assistance of Ms Ch. Heinze is gratefully acknowledged. Thanks are due to Ms A. Fischer for help in the preparation of the manuscript.

### REFERENCES

BERKE, G. (1980) Interaction of cytotoxic T lymphocytes and target cells. *Prog. Aller.*, **27**, 69.

BOSSLET, K. & SCHIRRMACHER, V. (1981) Escape of metastasizing clonal tumour cell variants from tumour-specific cytolytic T lymphocytes. *J. Exp. Med.*, **154**, 557.

HERBERMAN, R. B. (1980) *Natural Cell-Mediated Immunity against Tumours*. London: Academic Press.

HERBERMAN, R. B., NUNN, M. E. & LAVRIN, D. H. (1975) Natural cytotoxic reactivity of mouse lymphoid cells against syngeneic and allogeneic tumours. I. Distribution of reactivity and specificity. *Int. J. Cancer*, **16**, 216.

KIESSLING, R., KLEIN, E. & WIGZELL, H. (1975) Natural killer cells in mouse. I. Cytotoxic cells with specificity for mouse Moloney leukaemia cells. Specificity and distribution according to genotype. *Eur. J. Immunol.*, **5**, 112.

KORNBLITH, P. L., POLLOK, L. A., COAKHAM, H. B., QUINDLEN, E. A. & WOOD, W. C. (1979) Cytotoxic antibody responses in astrocytoma patients. An improved allogeneic assay. *J. Neurosurg.*, **51**, 47.

KURTH, R. & MEDLEY, G. (1975) A membrane permeability test for the detection of cell surface antigens. *Immunology*, **29**, 803.

LENEVA, N. V. & SVET-MOLDAVSKY, G. J. (1974) Susceptibility of tumour cells in different phases of the mitotic cycle to the effect of immune lymphocytes. *J. Natl Cancer Inst.*, **52**, 699.

MANTOVANI, A., ALLAVENA, P., SESSA, C., BOLIS, G. & MANGIONI, C. (1980) Natural killer activity of lymphoid cells isolated from human ascitic ovarian tumours. *Int. J. Cancer*, **25**, 573.

MCBRIDE, W. H. & BARD, J. B. L. (1979) Hyaluronidase-sensitive halos around adherent cells. Their role in blocking lymphocyte-mediated cytolysis. *J. Exp. Med.*, **149**, 507.

MCCORMICK, K. J., GIOVANELLA, B. C., KLEIN, G., NILSSON, K & STEHLIN, J. S. (1981) Diploid human lymphoblastoid and Burkitt lymphoma cell lines: Susceptibility to murine NK cells and heterotransplantation to nude mice. *Int. J. Cancer*, **28**, 455.

ORTALDO, J. R., OLDHAM, R. K., CANNON, G. C. & HERBERMAN R. B. (1977) Specificity of natural cytotoxic reactivity of normal human lymphocytes against a myeloid leukaemia cell line. *J. Natl Cancer Inst.*, **59**, 77.

PROSS, H. F. & BAINES, M. G. (1977) Spontaneous human lymphocyte-mediated cytotoxicity against tumour target cells. IV. A brief review. *Cancer Immunol. Immunother.*, **3**, 75.

SCHLAGER, S. I. (1981) Relationship between cell-mediated and humoral immune attack on tumour cells. I. Drug and hormone effects on susceptibility to killing and macro-molecular synthesis. *Cell. Immunol.*, **58**, 398.

SCHLAGER, S. I. & OHANIAN, S. H. (1979) Physical and chemical composition of subcellular fractions from tumour cells treated with metabolic inhibitors or hormones. *Cancer Res.*, **39**, 1369.

SCHLAGER, S. I. & OHANIAN, S. H. (1980) Tumour cell lipid composition and sensitivity to humoral immune killing. II. Influence of plasma membrane and intracellular lipid and fatty acid content. *J. Immunol.*, **125**, 508.

VOSE, B. M., VÀNKY, F. & KLEIN, E. (1977) Lymphocyte cytotoxicity against autologous tumour biopsy cells in humans. *Int. J. Cancer*, **20**, 512.

ZÄNKER, K. S. & BLÜMEL, G. (1981) Current concept on tumour cell stasis by inhibition of fibrin degradation. In *Progress in Fibrinolysis and Thrombolysis*. Vol. V, London: Churchill Livingstone, p. 242.

ZÄNKER, K. S., STAVROU, D., HULTÉN, M. & BILZER, TH. (1981*a*) The influence of various chemicals on the surface structure and the antigenicity of syngeneic glioma cells. *Anticancer Res.*, **1**, 101.

ZÄNKER, K. S., TRAPPE, A. & BLÜMEL, G. (1981*b*) *In-vitro* evaluation of the action of FOY II on tumour cell growth inhibition, *Eur. Surg. Res.*, **14**, 162.